# Tri-Ponderal Mass Index Reference Values for Screening Metabolic Syndrome in Children and Adolescents: Results From Two National-Representative Cross-Sectional Studies in China and America

**DOI:** 10.3389/fendo.2021.739277

**Published:** 2021-11-08

**Authors:** Xijie Wang, Yanjun Chen, Jun Ma, Bin Dong, Yanhui Dong, Zhiyong Zou, Yinghua Ma, Luke Arnold, Wannian Liang

**Affiliations:** ^1^ Vanke School of Public Health, Tsinghua University, Beijing, China; ^2^ Institute for Healthy China, Tsinghua University, Beijing, China; ^3^ Institute of Child and Adolescent Health & School of Public Health, Peking University, Beijing, China; ^4^ Clinical Nutrition Department, People’s Hospital of Ningxia Hui Autonomous Region Health Management Center, Yinchua, China; ^5^ Department of Commissioning, South Western Sydney Primary Health Network, Campbelltown, NSW, Australia

**Keywords:** tri-ponderal mass index, metabolic syndrome, child and adolescent health, health screening, cardiovascular risk

## Abstract

**Introduction:**

To ascertain the possible cut point of tri-ponderal mass index (TMI) in discriminating metabolic syndrome (MetS) and related cardio-metabolic risk factors in Chinese and American children and adolescents.

**Methods:**

A total of 57,201 Chinese children aged 7-18 recruited in 2012 and and 10,441 American children aged 12-18 from National Health and Nutrition Examination Survey (NHANES 2001-2014) were included to fit TMI percentiles. Participants were randomly assigned to a derivation set (75%) and validation set (25%). The cut points of TMI with the lowest misclassification rate under the premise of the highest area under curves (AUC) were selected for each sex, which were additionally examined in the validation set. All of data analysis was conducted between September and December in 2019.

**Results:**

TMI showed good capacity on discriminating MetS, with AUC of 0.7658 (95% CI: 0.7544-0.7770) to 0.8445 (95% CI: 0.8349-0.8537) in Chinese and 0.8871 (95% CI: 0.8663-0.9056) to 0.9329 (95% CI: 0.9166-0.9469) in American children. The optimal cut points were 14.46 kg/m^3^ and 13.91 kg/m^3^ for Chinese boys and girls, and 17.08 kg/m^3^ and 18.89 kg/m^3^ for American boys and girls, respectively. The corresponding misclassification rates were 17.1% (95% CI: 16.4-17.8) and 11.2% (95% CI: 9.9-12.6), respectively. Performance of these cut points were also examined in the validation set (sensitivity 67.7%, specificity 82.4% in Chinese; sensitivity 84.4%, specificity 88.7% in American children).

**Conclusions:**

A sex- and ethnicity- specific single cut point of TMI could be used to distinguish MetS and elevated risk of cardio-metabolic factors in children and adolescents.

## Introduction

Metabolic syndrome (MetS), specified by the National Cholesterol Education Program Adult Treatment Panel III (ATP III), is a strong and independent predictor of all-cause and cardiovascular disease (CVD) mortality (1, 2). Its prevalence has increased from approximately 2% in the mid-1990s to a current estimate of 10% in American children and adolescents (3–5). It was also estimated that nearly two thirds (63.4%) of American adolescents had at least 1 metabolic abnormality (5). In Chinese children aged 10-16, the prevalence of MetS ranged from 5.5% to 10.9%, depending on the definition used in the study (6, 7). Results from The China Health and Nutrition Survey suggested an overall prevalence of 3.37% in children aged 7-18, using ATP III definition (8). Since childhood MetS is likely to track into adulthood, early identification could help to prevent definitive lesions (9) and target interventions to improve future cardiovascular health (5, 10).

In consideration of the great importance of early identification, an accessible screening tool is needed for daily public health practice (11), especially in the less developed areas where professional pediatricians are deficient. Body mass index (BMI), calculated as weight(kg)/height^2^ (m^2^) has been the most widely used indicator in screening obesity and related diseases, including MetS (12). In recent decades, given the fact that central obesity is the biggest risk factor of MetS (13), waist circumference has been accepted as another efficient screening tool for MetS in young population (14–16). However, as children’s body shape changes dramatically during puberty, both measures required multiple cut-off values to fit age and sex subgroups of children.

Tri-ponderal mass index (TMI), calculated as weight(kg)/height^3^ (m^3^), has been reported to be a satisfactory adiposity indicator with good age-stability during adolescence (17, 18). Studies have also proven it to be an efficient indicator in screening obesity related cardio-metabolic risks including dyslipidemia, insulin resistance and other cardiovascular diseases (19–21). However, few studies have evaluated TMI cut point for screening of MetS and cardio-metabolic risks in pediatric populations. Although one study conducted in Colombian children and adolescents suggested that TMI could be a satisfying indicator to discriminate MetS, the authors gave age- and sex- specific screening thresholds (22), which didn’t take the best advantage of its age-stability.

Using cross-sectional baseline data from the Chinese school-based Health Life Intervention and United States National Health and Nutrition Examination Survey (NHANES 2001-2014), the present study aimed to ascertain the optimal TMI cut point for identifying MetS and cardio-metabolic risk factors in Chinese and American children and adolescents.

## Methods

### Study Population

Data for Chinese participants were obtained from baseline data of a Chinese school-based Health Life Intervention study, which was a national school-based multi-centered cluster randomized controlled trial against obesity in Chinese children and adolescents conducted in 2012. The sampling procedure of this study has been published elsewhere in detail (23). Briefly, more than 60,000 children and adolescents from 7 provinces, including Liaoning, Tianjin, Ningxia, Shanghai, Chongqing, Hunan, and Guangdong, participated in the study. In each province, 12-16 primary and secondary schools totaling around 10,000 participants were randomly selected. Of each selected school, two classes were randomly selected from each grade and students in those classes were invited for blood sample collection. Of the 59,916 participants aged 7 to 18 years, 2,715 were excluded due to missing sex, height or weight data. The remaining sample size for TMI derivation and analysis was 57,201, including 15,045 participants with blood samples. The original study was approved by Ethical Committee of the university. All participants and their parents provided signed informed consent.

Data for American participants were obtained from 7 cycles of National Health and Nutrition Examination Survey (NHANES 2001-2014) public release data, for which collection methods had been described in detail elsewhere (24). NHANES is an American national representative and continuous cross-sectional survey conducted every 2 years. In this study, only adolescents aged between 12 and 18 were included, as serum glucose and lipid profiles were unavailable for those younger than 12. Among those aged 12 to 18 years old, 667 participants with missing data of height and weight were excluded. Thus, the sample size for TMI analysis in this study was 10,441, and the sample size for the following analysis was 2,910.

Within each population, participants were randomly allocated to derivation set (75%) and validation set (25%) by a random number table in Stata. Participants with full records of height, weight and age were recruited in fitting TMI percentiles, while only those with complete records of all 5 risk components of MetS were recruited in the following analysis. Basically, the optimal TMI cut points were developed from derivation set and were validated in validation set.

### Measurements

All Chinese participants underwent physical examination according to a standard protocol (19). Height was measured using the portable stadiometer (model TZG, China) to the nearest 0.1 cm, with participants standing straight barefoot. Weight was measured with lever type weight scale (model RGT-140, China) to the nearest 0.1 kg, with participants wearing light underwear. Waist circumference was measured with steel tape at 1 cm above umbilicus to the nearest 1 mm. Height, weight and waist circumference were all measured twice and the mean values were recorded.

Blood pressure was measured according to the recommendation of the National High Blood Pressure Education Program (NHBPEP) Working Group in Children and Adolescents (21). Mercury sphygmomanometers (model XJ11D, China), stethoscopes (model TZ-1, China), and appropriate cuffs were used for blood pressure measurement. Participants were asked to sit quietly for at least 5 minutes prior to the first reading. Systolic blood pressure (SBP) was determined by onset of the first Korotkoff sound and diastolic blood pressure (DBP) was determined by the fifth Korotkoff sound. Blood pressure was measured twice with five minutes’ gap between two measurements, and the average of SBP and DBP values were separately calculated. Blood samples were collected after a 12-hour fasting. Fasting plasma glucose (FPG), triglyceride (TG), and high-density lipoprotein cholesterol (HDL-C) were measured using an automatic biochemical analyzer (Roche Modular P800 ISE900; Hoffmann-La Roche Ltd) by a qualified biological testing company. Rigid quality control was enforced in this study. All measurement instruments were calibrated before use, and all examiners were required to pass a standard training course before commencement.

For American participants, anthropometric measurements were collected according to protocols previously published in detail (24). Sex and age were self-reported during home interviews. FPG, TG and HDL-C levels were measured enzymatically at Johns Hopkins University Lipoprotein Analytic Laboratory with the use of a Hitachi 704 Analyzer (Boehringer Mannheim Diagnostics, Indianapolis, IN). Lipid collection and analyses were standardized to Centers for Disease Control and Prevention criteria (25).

TMI for each participant was calculated as weight (kg)/height^3^ (m^3^).

### Definitions of Metabolic Syndrome

According to the criteria of National Cholesterol Education Program (NCEP) Adult Treatment Panel III (ATP III), children were considered to have MetS if they showed three or more of the following (2, 26): (1) central obesity (waist circumference ≥ 90^th^ sex- and age-specific percentile); (2) high fasting glucose (FPG ≥ 110 mg/dL or 6.1 mmol/L); (3) hypertriglyceridemia (TG ≥ 110 mg/dL or 1.242 mmol/L); (4) low HDL-C (HDL-C ≤ 40 mg/mL or 1.0344 mmol/L); (5) high blood pressure (blood pressure ≥ 90^th^ sex-, age- and height-specific percentile). The definitions were also showed in **Supplementary Table 1**.

As reference values for waist circumference vary across ethnic groups, “High Waist Circumference Screening Threshold Among Chinese Children and Adolescents Aged 7-18 years” (27) and “Anthropometric Reference Data for Children and Adults: United States” (28) were used for Chinese and American populations, respectively. Blood pressure was evaluated with the 2017 version of the Clinical practice guideline for screening and management of high blood pressure in children and adolescents (29).

### Statistical Analysis

All of data analysis was conducted between September and December in 2019. The characteristics of participants were given as mean (SD) or number (%). Independent two-tailed t-tests for continuous variables and chi-square (χ^2^) tests for categorical variables were used to compare the differences between derivation set and validation set in Chinese and American participants, respectively. LMS curves were generated with LMScharmaker (version 2.5, developed by HARLOW PRINTING LIMITED) to estimate TMI changes with age in both sexes in Chinese and American participants, respectively. Since TMI showed good stability with increasing age, especially within the range from P_5_ to P_90_ (results displayed in **Supplementary Figure 1**), the following analyses were carried in the absence of age factors.

The relationships between TMI percentile and prevalence of MetS components were estimated with logistic regression and fractional polynomial regression models by sex. Receiver operating characteristic (ROC) analysis was used to find the possible range of TMI cut-off percentiles, and the alternative points were identified as those with the highest Youden Index (30). Five optimal cut-off percentiles were selected for further analysis based on their capacity to distinguish MetS. The cut-off percentile with the lowest misclassification rate were selected under the premise of the largest area under ROC curves (AUC) (31). The cut-off values obtained from the derivation set were then validated in the validation set, by calculating their AUC and misclassification rate. Sensitivity analyses were conducted between sex groups for participants in both countries, while urban-rural disparities and ethical disparities were estimated for Chinese and American populations, respectively. All analyses were performed using Stata 14.0 (College Station, TX, USA) and associations were considered significant when performed at levels of *P* < 0.05 (two sides).

## Results

A total of 57,201 Chinese participants and 10,441 American participants were included in the present study, while 15,045 Chinese and 2,910 American participants had complete records of MetS risk components. The summary of their characteristics, including height, weight, waist circumference and MetS risk factors, are shown in **Table 1**. The prevalence of MetS is 6.6% to 7.0% for Chinese participants and 3.7% to 4.3% for American participants depending on derivation or validation set. Generally, no significant difference in MetS prevalence or other variables was observed between the two sets.

**Table 1:** Characteristics of Chinese and American participants, with mean and standard deviation (SD) for continuous variable and number and % for categorical variables.

Characteristics	Chinese population 1		Chinese population 2		*p value*	American population 1		American population 2		*p value*
	N	Data	N	Data		N	Data	N	Data	
Development of TMI percentiles										
Age, mean (SD), year	42,901	11.3 (3.1)	14,300	11.3 (3.1)	0.718	7,830	15.0 (2.0)	2611	14.9 (2.0)	0.004^**^
Boys, number (%)	42,901	22,117 (51.6)	14,300	7374 (51.6)	0.979	7,830	4009 (51.2)	2611	1305 (50.0)	0.280
Height, mean (SD), cm	42,901	148.2 (16.0)	14,300	148.3 (16.0)	0.818	7,830	164.9 (10.0)	2611	164.5 (10.2)	0.072
Weight, mean (SD), kg	42,901	42.6 (15.1)	14,300	42.8 (15.4)	0.107	7,830	65.0 (19.3)	2611	65.4 (20.3)	0.382
Analysis for metabolic syndrome										
Waist circumference, mean (SD), cm	11,302	65.9 (10.7)	3743	66.1 (10.9)	0.382	2159	80.6 (14.1)	751	81.3 (14.9)	0.309
TG, mean (SD), mmol/L	11,302	1.16 (0.82)	3743	1.18 (0.84)	0.176	2159	0.96 (0.58)	751	0.94 (0.56)	0.398
HDL-C, mean (SD), mmol/L	11,302	1.91 (1.38)	3743	1.92 (1.39)	0.787	2159	1.37 (0.32)	751	1.35 (0.32)	0.283
FPG, mean (SD), mmol/L	11,302	4.12 (1.30)	3743	4.10 (1.33)	0.786	2159	5.11 (0.83)	751	5.13 (1.09)	0.616
SBP, mean (SD), mmHg	11,302	104.6 (12.0)	3743	105.0 (11.9)	0.161	2159	108.9 (10.1)	751	109.1 (10.2)	0.681
DBP, mean (SD), mmHg	11,302	66.5 (9.0)	3743	66.5 (8.8)	0.944	2159	59.6 (11.3)	751	59.6 (10.8)	0.954
Central obesity^†^, number (%)	11,302	2430 (21.5)	3743	872 (23.3)	0.021^*^	2159	172 (8.0)	751	76 (10.1)	0.069
Dyslipidemia (TG), number (%)	11,302	3019 (26.7)	3743	1065 (28.5)	0.038^*^	2159	441 (20.4)	751	142 (18.9)	0.371
Dyslipidemia (HDL-C), number (%)	11,302	1421 (12.6)	3743	512 (13.7)	0.080	2159	309 (14.3)	751	131 (17.4)	0.039^*^
Glucose intolerance, number (%)	11,302	32 (0.28)	3743	10 (0.27)	0.872	2159	45 (2.1)	751	16 (2.1)	0.939
High blood pressure, number (%)	11,302	2487 (22.0)	3743	828 (22.1)	0.882	2159	158 (7.3)	751	61 (8.1)	0.472
Metabolic syndrome, number (%)	11,302	752 (6.6)	3743	263 (7.0)	0.431	2159	79 (3.7)	751	32 (4.3)	0.458

TG, triglyceride; HDL-C, high density lipoprotein cholesterol; FPG, fasting plasma glucose; SBP, systolic blood pressure; DBP, diastolic blood pressure.

^†^Central obesity was defined as waist circumference ≥ 90th percentile (age- and sex- specific) for Chinese and American populations, respectively.

^*^P < 0.05, ^**^P < 0.01.

**Figure 1:**
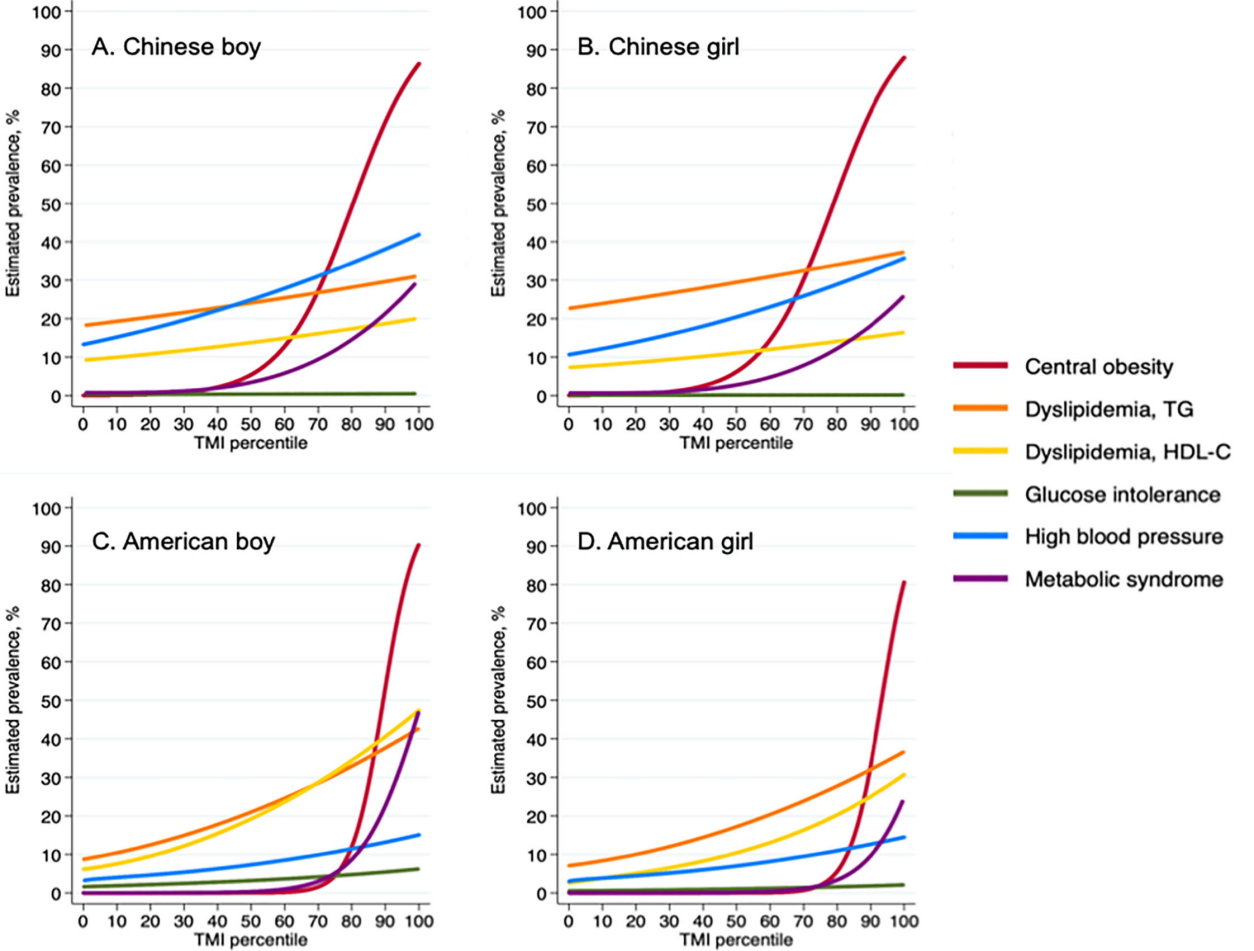
Prevalence of cardiometabolic risk factors according to Tri-ponderal mass index percentiles among Chinese and American children. TMI, tri-ponderal mass index (kg/m^3^); TG, triglyceride; HDL-C, high density lipoprotein cholesterol; FPG, fasting plasma glucose. **(A)** Chinese boy; **(B)** Chinese girl; **(C)** American boy; **(D)** American girl.

As TMI percentile increased, estimated prevalence of all cardio-metabolic risk factors, including MetS, increased considerably. Notably, between P_70_ and P_80_ in Chinese participants, the estimated prevalence of MetS increased rapidly in both sexes. A similar pattern was also detected in American participants, notably between P_80_ and P_90_ (**Figures 1A–D**).

TMI showed good capacity in diagnosing MetS in both Chinese and American participants. The area under ROC curves for TMI-MetS were 0.8445 (95% CI: 0.8349-0.8537, boys) and 0.7658 (95% CI: 0.7544-0.7770, girls) for Chinese participants and 0.9329 (95% CI: 0.9166-0.9469, boys) and 0.8871 (95% CI: 0.8663-0.9056, girls) for American participants. The optimal cut-off percentiles ranged from 72.9 to 79.9 based on the highest Youden index in Chinese and American participants (**Supplementary Table 2**), therefore P_70_, P_75_, P_80_, P_85_ and P_90_ were selected for further evaluation. The corresponding TMI values of these percentiles are listed in **Supplementary Table 3**.

**Figure 2:**
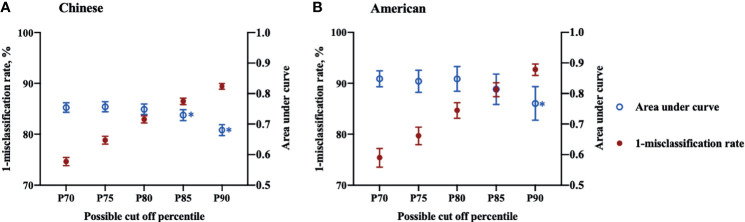
Misclassification rates and area under curves of Tri-ponderal mass index in assessing metabolic syndrome in Chinese and American children. Sex specific P_70_, P_75_, P_80_, P_85_ and P_90_ were used as threshold values for each population.* The area under curve decreased significantly compared to the former cut off percentile. **(A)** Chinese children; **(B)** American children.

The results of AUC and misclassification rate analyses are shown in **Figures 2A, B**, demonstrating a reduction in the misclassification rate and AUC as higher percentiles were tested. In Chinese participants, AUC of P_70_, P_75_ and P_80_ varied from 0.7486 (95% CI: 0.7313-0.7660) to 0.7570 (95% CI: 0.7405-0.7735) with no statistical differences, while the AUC decreased significantly from P_80_ (0.7570, 95% CI: 0.7405-0.7735) to P_85_ (0.7294, 95% CI: 0.7115-0.7474). In American participants, the inflection point of AUC was around P_90_, where the AUC declined significantly from 0.8140 (95% CI: 0.7645-0.8634) for P_85_ to 0.7674 (95% CI: 0.7127-0.8221). Under the premise of keeping the highest AUC, the points with the lowest misclassification rate were selected for Chinese and American participants, respectively, as the possible thresholds for discriminating MetS (31). The corresponding misclassification rates were 17.1% (95% CI: 16.4-17.8) and 11.2% (95% CI: 9.9-12.6) in Chinese and American populations, respectively. Therefore, the optimal TMI percentile was P_80_ (TMI values of 14.46 kg/m^3^ for males and 13.91 kg/m^3^) for females, respectively) for Chinese participants and P_85_ (TMI values of 17.08 kg/m^3^ for males and 18.89 kg/m^3^ for females, respectively) for American participants. The selected optimal TMI values were additionally tested in the validation set, and the results supported what was found in the derivation set (**Table 2**).

**Table 2:** Validation of selected cut-off percentile of Tri-ponderal mass index in identifying cardiometabolic risks in Chinese and American validate populations.

Population	Area Under Curve	FPR	FNR	TFR	Sensitivity, %	Specificity, %
Chinese						
P_75_	0.7624 (0.7355, 0.7893)	23.2 (21.8, 24.6)	24.3 (19.3, 30.0)	23.3 (21.9, 24.7)	75.7 (70.1, 80.5)	76.8 (75.4, 78.2)
P_80_	0.7502 (0.7212, 0.7792)	17.6 (16.4, 19.0)	32.3 (26.7, 38.3)	18.7 (17.4, 20.0)	67.7 (61.8, 73.0)	82.4 (81.1, 83.6)
P_85_	0.7322 (0.7177, 0.7463)^†^	12.5 (11.4, 13.6)	41.1 (35.1, 47.3)	14.5 (13.4, 15.7)	58.9 (52.9, 64.7)	87.5 (86.4, 88.6)
American						
P_80_	0.8763 (0.8314, 0.9212)	18.5 (15.7, 21.5)	6.7 (0.8, 22.1)	18.0 (15.3, 20.9)	93.8 (79.9, 98.3)	81.5 (78.5, 84.2)
P_85_	0.8656 (0.839, 0.8891)	11.3 (9.0, 13.8)	15.6 (5.3, 32.8)	11.5 (9.3, 13.9)	84.4 (68.2, 93.1)	88.7 (86.2, 90.8)
P_90_	0.8048 (0.7226, 0.887)^†^	7.8 (5.9, 10.0)	31.3 (16.1, 50)	8.8 (6.9, 11.0)	68.8 (51.4, 82.0)	92.2 (90, 94.0)

FPR, false positive rate; FNR, false negative rate; TR, total misclassification rate.

^†^Area under curve decreased significantly compared to the former cut off percentile.

The results for sensitivity analyses were displayed in **Supplementary Tables 4**, **5** with derivation population. In Chinese population, the capacity of the cut points were generally consistent with those from all population, although 13.93 kg/m^3^ (P_75_) may be optimal for Chinese boys. However, as the sample size of derivations set was rather small, the results should be test in further studies with bigger population. In the American population, P_85_ showed good consistency in all participants except for Mexican American, for whom P_90_ may be more appropriate cut-off percentile, as the AUC was significantly higher than other percentiles.

## Discussion

MetS affects approximately 35% of adults in the United States of America and is also a great threat in developing countries, including China (32, 33). Let alone the MetS and related cardiovascular risks originated from childhood has significant influence on future diseases (34), it is of consensus that regular monitoring/management is important for children, especially for those with high MetS risk (7).

The present study found that TMI could be a satisfying index in identifying MetS and other cardio-metabolic risks in young Chinese and American populations. The thresholds established to identify elevated MetS risk were 14.46 kg/m^3^ for boys *vs*. 13.91 kg/m^3^ for girls in Chinese participants aged between 7 to 18, and 17.08 kg/m^3^ for boys *vs*. 18.89 kg/m ^3^ for girls in American participants aged between 12 to 18. These cut points showed favorable capacity in discriminating MetS and related risks and performed good consistency in subgroup analysis.

TMI as an alternative option for BMI has been paid increasing attention in recent years, especially in pediatric populations where BMI changes with age. Previous studies found that TMI remained steady throughout childhood and showed good consistency to the percent of body fat (17, 18, 20). These qualities made TMI a satisfactory indicator in daily screening of excessive weight and related cardiometabolic diseases. The present study verified its age-stability and ulterior discussed the possible TMI thresholds to discriminate MetS in both Chinese and American children. Compared to the thresholds derived from a previous study in a Columbian population (22), the present study found higher TMI cut-points in Chinese children, and even higher cut-points in American children. The distinction between ethnic groups had been discovered previously when researchers attempted to set country-specific BMI thresholds, as they found that people from Asia and Pacific regions had a higher percent of body fat and waist-to-hip ratio than Caucasians at a given BMI (35). Further studies also found that even if their anthropometric measurements were within a safe range, people from eastern countries would have higher disease risk than their western counterparts (36, 37). Besides, various cut-off points among different sex, race and ethic subgroups were found, indicating that even if TMI presented good age-stability during childhood, sex and ethic disparities should be considered in setting country-specific cut-points. For more validated TMI cutoff points, more standardized research under global cooperation should be conducted, in order to eliminate any possible deviations resulted from operators and testing examinations. For many years, researchers have been seeking an appropriate screening tool to identify adiposity, as well as obesity-related disease risk in children. Apart from age- and sex- specific BMI used for screening general adiposity, waist circumference and waist-to-height ratio are also becoming popular for central obesity (38). However, as these indicators were normally recommended for further assessment in children with risk of developing other long-term health problems than obesity, their applications in general younger populations were still under debate (39). Although, in our previous research, further combination with waist circumference did not improve the ability of discriminating high risk population of TMI in children and adolescents (20). The present study, with two national-representative surveys, provided a possible choice with both age-stability and good capacity in recognizing cardio-metabolic abnormalities. From a practical point of view, TMI bases only on height and weight measurements, and requires very simple calculation. The number of TMI thresholds for screening adiposity is also much less than those of BMI, and doesn’t depend on age- specific percentiles. These qualities make TMI an accessible, easy, and accurate indicator for long-term monitoring of childhood cardio-metabolic abnormalities in primary care settings. And it could also be useful for school-based and community surveillance efforts in early prevention of cardio-metabolic risks (40).

The current study had its limitations. Cross-sectional data was obtained from Chinese and American populations. Although majority of adolescent years were included in the present study, TMI variance at individual levels was still undetected. Meanwhile, it remained undetected that whether the time lag between two populations would make any difference to the primary findings of the present study. In addition, ethnic differences must be taken into consideration in application of TMI. Although this study conducted ethnic-stratified sensitivity analyses in American participants and found good consistency between non-Hispanic white, non-Hispanic black and other ethnic groups, it may be insufficient for making a general cut-point. Ethnic-specific studies may need further consideration, while any differences resulted from operator, testing equipment or definition on specific metabolic disorders should also be taken into consideration. Moreover, the method of waist circumference in Chinese children was not standard. Although it was proved to be similar to the standard method in analyzing abdominal fat (data published in Chinese only), underestimation of actual waist circumference was also possible. This emphasizes the importance of collaborative and standardized cooperation again.

TMI could be an accurate and convenient population-based screening tool for MetS and related cardio-metabolic risk factors in Chinese and American children and adolescents. Corresponding TMI values for sex-specific P_80_ in Chinese and P_85_ in American populations could be utilized as appropriate thresholds in clinical practice.

## Data Availability Statement

The raw data supporting the conclusions of this article will be made available by the authors, upon reasonable request.

## Ethics Statement

The studies involving human participants were reviewed and approved by Ethical Committee of Peking University. Written informed consent to participate in this study was provided by the participants’ legal guardian/next of kin.

## Author Contributions

XW performed all statistical analyses and wrote the manuscript. JM is the principle investigator of the original study. BD and YD are co-investigators and reviewed and edited the manuscript. ZZ and YM are co-investigator of the original study. LA, YC, and WL reviewed and edited the manuscript. XW takes full responsibility for the contents of the article. All authors contributed to the article and approved the submitted version.

## Funding

The present research was supported by funding of National Natural Science Foundation of China (No. 81673192, rewarded to JM; No. 81903344, rewarded to BD), Young Researcher Personal Project of Beijing to BD.

## Conflict of Interest

The authors declare that the research was conducted in the absence of any commercial or financial relationships that could be construed as a potential conflict of interest.

## Publisher’s Note

All claims expressed in this article are solely those of the authors and do not necessarily represent those of their affiliated organizations, or those of the publisher, the editors and the reviewers. Any product that may be evaluated in this article, or claim that may be made by its manufacturer, is not guaranteed or endorsed by the publisher.

## Supplementary Material

Supplementary Figure 1 Age- and sex- specific Tri-ponderal mass index (TMI) values for Chinese children aged 7 to 18 and for American children aged 12 to 18.

Supplementary Table 1 Definition of cardiometabolic risk factors. Waist circumference percentiles were determined from “High waist circumference screening threshold among children and adolescents aged 7–18 years” for Chinese children, and from Anthropometric reference data for children and adults: United States, 2011-2014 for American children.

Supplementary Table 2 Parameters of the receiver operating characteristic (ROC) curves analysis for the diagnostic performance of Tri-ponderal Mass (TMI) in identifying cardiometabolic risks in Chinese and American children. TG, triglycerides; HDL-C, high density lipoprotein cholesterol; CI, confidential interval; PV (+), positive predictive value; PV (-), negative predictive value.

Supplementary Table 3 Tri-ponderal mass index threshold for the optimal cut-off percentiles.

Supplementary Table 4 Sensitivity analysis of different subgroups in Chinese validate population. FPR, false positive rate; FNR, false negative rate; TR, total misclassification rate. * Area under curve decreased significantly compared to the former cut off percentile. Sex specific 75^th^, 80^th^ and 85^th^ percentiles were used as threshold values for each population.

Supplementary Table 5 Sensitivity analysis of different subgroups in American validate population.
