# Supplementary material for: Tri-Ponderal Mass Index Reference Values for Screening Metabolic Syndrome in Children and Adolescents: Results From Two National-Representative Cross-Sectional Studies in China and America
Source: Front Endocrinol (Lausanne). 2021 Nov 8;12:739277. doi: 10.3389/fendo.2021.739277 (PMC8606676; doi:10.3389/fendo.2021.739277)
Supplement: Supplementary Figure 1 — Age- and sex- specific Tri-ponderal mass index (TMI) values for Chinese children aged 7 to 18 and for American children aged 12 to 18. [file Image_1.pdf]

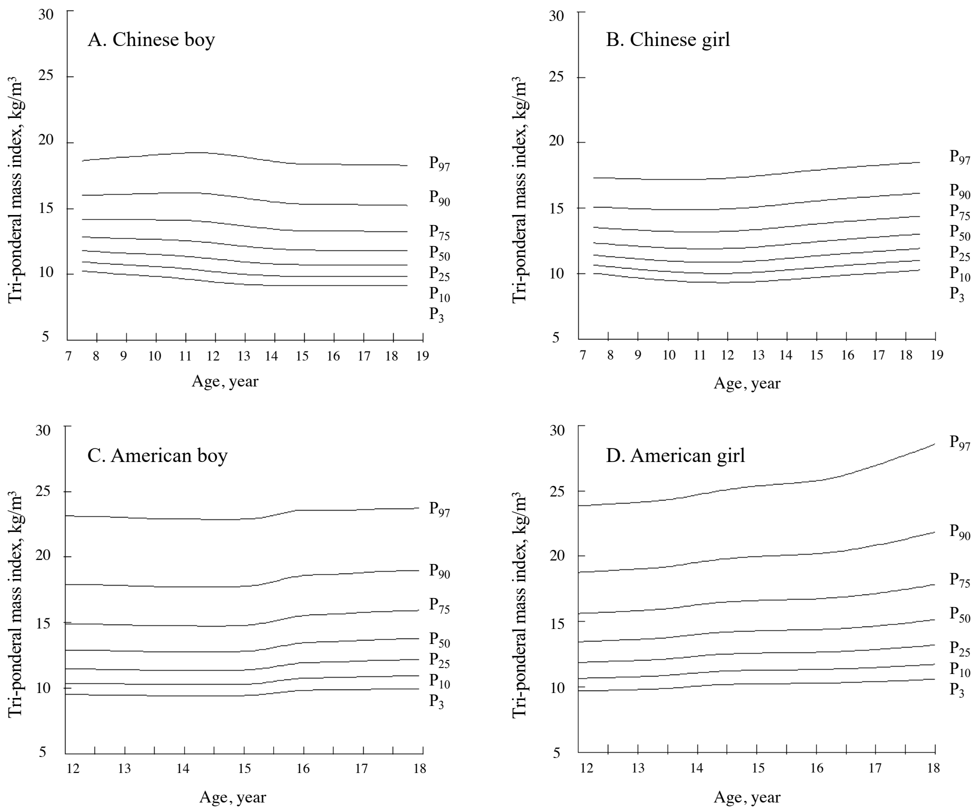

S. Figure 1. Age- and sex- specific Tri-ponderal mass index (TMI) values for Chinese children aged 7 to 18 and for American children aged 12 to 18.
