# Supplementary material for: Tri-Ponderal Mass Index Reference Values for Screening Metabolic Syndrome in Children and Adolescents: Results From Two National-Representative Cross-Sectional Studies in China and America
Source: Front Endocrinol (Lausanne). 2021 Nov 8;12:739277. doi: 10.3389/fendo.2021.739277 (PMC8606676; doi:10.3389/fendo.2021.739277)
Supplement: Supplementary Table 1 — Definition of cardiometabolic risk factors. Waist circumference percentiles were determined from “High waist circumference screening threshold among children and adolescents aged 7–18 years” for Chinese children, and from Anthropometric reference data for children and adults: United States, 2011-2014 for American children. [file Table_1.docx]

| Supplementary Table 1. Definition of cardiometabolic risk factors | | |
| --- | --- | --- |
| Component | | Definition |
| Central obesity | | Waist circumference ≥ 90th percentile (age and sex specific)* |
| Dyslipidemia | Triglycerides | ≥ 1.2419 mmol/L (110 mg/dL) |
|  | HDL-C | ≤ 1.03 mmol/L |
| Glucose intolerance | | Fasting plasma glucose ≥ 6.1mmol/L |
| High blood pressure | | Blood pressure ≥ 90th percentile (age, sex, and height specific) |
| Metabolic syndrome | | ≥ 3 of above 5 indicators |
| Waist circumference percentiles were determined from “High waist circumference screening threshold among children and adolescents aged 7～18 years” for Chinese children, and from Anthropometric reference data for children and adults: United States, 2011-2014 for American children. | | |
|  |  |  |
|  |  |  |
|  |  |  |
|  |  |  |

| Supplementary Table 2. Parameters of the receiver operating characteristic (ROC) curves analysis for the diagnostic performance of Tri-ponderal Mass (TMI) in identifying cardiometabolic risks in Chinese and American children. | | | | | | | | | | | | |
| --- | --- | --- | --- | --- | --- | --- | --- | --- | --- | --- | --- | --- |
| Parameter | Obesity | | Dyslipidemia (TG) | | Dyslipidemia (HDL-C) | | Glucose intolerance | | High blood pressure | | Metabolic syndrome | |
|  | Boys | Girls | Boys | Girls | Boys | Girls | Boys | Girls | Boys | Girls | Boys | Girls |
| **Chinese population** |  |  |  |  |  |  |  |  |  |  |  |  |
| Area under curve | 0.9470 | 0.8897 | 0.5707 | 0.5460 | 0.5597 | 0.5941 | 0.4915 | 0.6888 | 0.6445 | 0.6001 | 0.8445 | 0.7658 |
| 95% CI | 0.9440,  0.9500 | 0.8854,  0.8940 | 0.5579,  0.5834 | 0.5328,  0.5592 | 0.5469,  0.5724 | 0.5810,  0.6071 | 0.4786,  0.5044 | 0.6763,  0.7010 | 0.6382,  0.6508 | 0.5934,  0.6068 | 0.8349,  0.8537 | 0.7544,  0.7770 |
| Cut-off (value) | 13.67 | 13.09 | 12.93 | 12.16 | 12.35 | 12.56 | 11.93 | 12.90 | 12.70 | 12.58 | 13.84 | 13.26 |
| Cut-off (percentile) | 72.1 | 68.0 | 62.1 | 48.1 | 51.1 | 57.4 | 42.5 | 64.3 | 58.4 | 57.9 | 74.1 | 72.9 |
| Sensitivity, % | 87.7 | 79.6 | 45.8 | 56.0 | 54.7 | 53.3 | 62.5 | 62.5 | 57.6 | 53.0 | 79.1 | 69.7 |
| Specificity, % | 88.2 | 81.8 | 67.1 | 50.4 | 54.4 | 59.5 | 44.1 | 65.0 | 63.9 | 60.7 | 79.9 | 73.7 |
| PV (+), % | 66.6 | 55.9 | 30.6 | 32.2 | 16.2 | 14.4 | 0.5 | 0.3 | 35.7 | 26.8 | 23.0 | 14.9 |
| PV (-), % | 96.4 | 93.3 | 79.6 | 73.1 | 88.1 | 90.0 | 99.7 | 99.9 | 81.2 | 82.6 | 98.0 | 97.4 |
| **American population** |  |  |  |  |  |  |  |  |  |  |  |  |
| Area under curve | 0.9780 | 0.9567 | 0.6933 | 0.6196 | 0.7105 | 0.6835 | 0.6204 | 0.5760 | 0.6309 | 0.6266 | 0.9329 | 0.8871 |
| 95% CI | 0.9730,  0.9824 | 0.9497,  0.963 | 0.6698,  0.7162 | 0.5937,  0.6450 | 0.6954,  0.7252 | 0.6676,  0.6990 | 0.5974,  0.6430 | 0.5515,  0.6002 | 0.6131,  0.6485 | 0.6085,  0.6445 | 0.9166,  0.9469 | 0.8663,  0.9056 |
| Cut-off (value) | 16.74 | 18.50 | 13.78 | 13.79 | 14.21 | 14.95 | 13.51 | 15.90 | 13.93 | 14.72 | 16.21 | 16.45 |
| Cut-off (centile) | 83.0 | 83.7 | 59.4 | 47.4 | 64.5 | 60.4 | 56.6 | 69.2 | 61.4 | 57.9 | 79.9 | 73.0 |
| Sensitivity, % | 93.3 | 90.4 | 63.7 | 64.3 | 61.7 | 65.7 | 61.7 | 50.0 | 56.8 | 60.0 | 89.7 | 90.5 |
| Specificity, % | 92.4 | 90.0 | 70.7 | 53.2 | 72.0 | 64.5 | 59.8 | 71.5 | 62.6 | 60.4 | 85.6 | 76.1 |
| PV (+), % | 60.2 | 42.5 | 38.0 | 23.4 | 37.9 | 20.9 | 5.1 | 1.9 | 11.7 | 11.1 | 25.5 | 7.2 |
| PV (-), % | 99.1 | 99.1 | 87.4 | 87.0 | 87.2 | 93.0 | 97.8 | 99.2 | 94.3 | 94.8 | 99.3 | 99.7 |
| Abbreviations: TG, triglycerides; HDL-C, high density lipoprotein cholesterol; CI, confidential interval; PV (+), positive predictive value; PV (-), negative predictive value. | | | | | | | | | | | | |

| Supplementary Table 3. Tri-ponderal mass index threshold for the optimal cut-off percentiles. | | | | | |
| --- | --- | --- | --- | --- | --- |
| Percentiles | Chinese | |  | American | |
|  | Boys | Girls |  | Boys | Girls |
| P1 | 9.19 | 9.30 |  | 9.39 | 9.72 |
| P50 | 12.27 | 12.24 |  | 12.99 | 13.97 |
| P70 | 13.48 | 13.2 |  | 14.82 | 16.05 |
| P75 | 13.93 | 13.52 |  | 15.35 | 16.73 |
| P80 | 14.46 | 13.91 |  | 16.22 | 17.62 |
| P85 | 15.12 | 14.43 |  | 17.08 | 18.89 |
| P90 | 15.98 | 15.15 |  | 18.39 | 20.12 |
| P99 | 19.68 | 18.98 |  | 25.17 | 27.49 |

| Supplementary Table 4. Sensitivity analysis of different subgroups in Chinese validate population. | | | | | |
| --- | --- | --- | --- | --- | --- |
| Subgroups | Cut-off threshold | Area under curve | FPR | FNR | TFR |
| Sex |  |  |  |  |  |
| Boy | P75 | 0.7629 (0.7263, 0.7996) | 22.6 (20.6, 24.6) | 24.8 (18.0, 32.7) | 22.8 (20.9, 24.7) |
|  | P80 | 0.7371 (0.6971, 0.7771)* | 17.4 (15.7, 19.3) | 35.2 (27.4, 43.5) | 18.8 (17.0, 20.6) |
|  | P85 | 0.7290 (0.6880, 0.7700)* | 12.8 (11.3, 14.5) | 41.4 (33.3, 49.8) | 15.0 (13.4, 16.7) |
| Girl | P75 | 0.7624 (0.7226, 0.8022) | 23.8 (21.8, 25.9) | 23.7 (16.4, 32.4) | 23.8 (21.9, 25.8) |
|  | P80 | 0.7666 (0.7246, 0.8086) | 17.9 (16.1, 19.8) | 28.8 (20.8, 37.9) | 18.6 (16.8, 20.4) |
|  | P85 | 0.7357 (0.6905, 0.7809)* | 12.2 (10.7, 13.8) | 40.7 (31.7, 50.1) | 14.0 (12.4, 15.7) |
| Region |  |  |  |  |  |
| Urban | P75 | 0.7479 (0.7134, 0.7824) | 22.4 (20.7, 24.2) | 28.0 (21.5, 35.3) | 22.8 (21.2, 24.6) |
|  | P80 | 0.7385 (0.7021, 0.7749) | 16.9 (15.3, 18.5) | 35.4 (28.4, 43.0) | 18.3 (16.7, 19.9) |
|  | P85 | 0.7120 (0.6744, 0.7497)* | 11.9 (10.5, 13.3) | 45.7 (38.2, 53.4) | 14.4 (13.0, 15.9) |
| Rural | P75 | 0.7925 (0.7514, 0.8337) | 24.4 (22.1, 26.9) | 17.0 (9.9, 26.6) | 24.0 (21.8, 26.3) |
|  | P80 | 0.7748 (0.7274, 0.8221) | 18.9 (16.8, 21.1) | 26.1 (17.3, 36.6) | 19.4 (17.3, 21.5) |
|  | P85 | 0.7733 (0.7235, 0.8231) | 13.5 (11.7, 15.5) | 31.8 (22.3, 42.6) | 14.7 (12.9, 16.6) |
| FPR, false positive rate; FNR, false negative rate; TR, total misclassification rate. * Area under curve decreased significantly compared to the former cut off percentile.  Sex specific 75^th^, 80^th^ and 85^th^ percentiles were used as threshold values for each population. | | | | | |
|  |  |  |  |  |  |

| Supplementary Table 5. Sensitivity analysis of different subgroups in American validate population. | | | | | |
| --- | --- | --- | --- | --- | --- |
| Subgroups | Cut-off threshold | Area under curve | FPR, % | FNR | TFR |
| Sex |  |  |  |  |  |
| Boy | P80 | 0.9066 (0.8870, 0.9262) | 18.7 (14.9, 23.0) | - | 18.0 (14.3, 22.1) |
|  | P85 | 0.8807 (0.7903, 0.9711) | 10.5 (7.6, 14.1) | 13.3 (1.7, 40.5) | 10.6 (7.8, 14.1) |
|  | P90 | 0.8338 (0.7173, 0.9503) | 6.6 (4.3, 9.6) | 26.7 (7.8, 55.1) | 7.3 (5.0, 10.4) |
| Girl | P80 | 0.8497 (0.7682, 0.9313) | 18.3 (14.3, 22.8) | 11.8 (1.5, 36.4) | 18.0 (14.1, 22.4) |
|  | P85 | 0.8513 (0.7563, 0.9463) | 12.1 (8.8, 16.0) | 17.6 (3.8, 43.4) | 12.4 (9.1, 16.2) |
|  | P90 | 0.7778 (0.6597, 0.8959) | 9.1 (6.3, 12.7) | 35.3 (14.2, 61.7) | 10.4 (7.4, 14.0) |
| Race |  |  |  |  |  |
| White | P80 | 0.8378 (0.7249, 0.9508) | 15.8 (11, 21.5) | 16.7 (2.1, 48.4) | 15.8 (11.2, 21.4) |
|  | P85 | 0.8773 (0.7656, 0.9889) | 7.9 (4.6, 12.5) | 16.7 (2.1, 48.4) | 8.4 (5.0, 12.9) |
|  | P90 | 0.7229 (0.5744, 0.8715)* | 5.4 (2.7, 9.5) | 50.0 (21.1, 78.9) | 7.9 (4.7, 12.4) |
| Black | P80 | 0.9059 (0.8789, 0.9330) | 18.8 (13.7, 24.9) | - | 18.4 (13.4, 24.4) |
|  | P85 | 0.8106 (0.5646, 1.0000) | 12.9 (8.6, 18.3) | 25.0 (0.6, 80.6) | 13.1 (8.8, 18.5) |
|  | P90 | 0.6980 (0.4143, 0.9817) | 10.4 (6.6, 15.5) | 50.0 (6.8, 93.2) | 11.2 (7.2, 16.3) |
| Mexican | P80 | 0.8859 (0.8572, 0.9146) | 22.8 (17.3, 29.2) | - | 21.6 (16.3, 27.6) |
|  | P85 | 0.8511 (0.7386, 0.9637) | 13.1 (8.8, 18.5) | 16.7 (2.1, 48.4) | 13.3 (9.1, 18.5) |
|  | P90 | 0.8803 (0.7687, 0.9918)* | 7.3 (4.1, 11.7) | 16.7 (2.1, 48.4) | 7.8 (4.6, 12.2) |
| Other | P80 | 0.9259 (0.8923, 0.9596) | 14.8 (8.7, 22.9) | - | 14.3 (8.4, 22.2) |
|  | P85 | 0.9444 (0.9147, 0.9742) | 11.1 (5.9, 18.6) | - | 10.7 (5.7, 18.0) |
|  | P90 | 0.9583 (0.9322, 0.9845) | 8.3 (3.9, 15.2) | - | 8.0 (3.7, 14.7) |
|  | | | | | |
